# High frequency ultrasound‐guided pericardiocentesis performed in the sitting position: A novel apical approach

**DOI:** 10.1002/clc.23657

**Published:** 2021-06-08

**Authors:** Lei Zhang, Xue‐Fei Zhang, Zhao Liu, Ying Liu, Cun‐Li Guo, Hua Shao, Bo Li, Cui Zhang, Hui Jing, Wen Cheng

**Affiliations:** ^1^ Department of Ultrasound Harbin Medical University Cancer Hospital Harbin China; ^2^ Interventional Ultrasound Ward Harbin Medical University Cancer Hospital Harbin China

**Keywords:** apical approach, high frequency ultrasound, pericardiocentesis, malignant pericardial effusion, percutaneous, sitting position

## Abstract

**Background:**

So far, few approaches have been described to reduce inadvertent injury to structure of the heart and nearby organs in percutaneous pericardiocentesis.

**Hypothesis:**

We hypothesized that an in‐plane high frequency ultrasound‐guided apical approach, performed in the sitting position, would provide additional benefits in terms of feasibility and safety for draining malignant pericardial effusion (MPE).

**Methods:**

The authors selected 53 consecutive patients with moderate or large symptomatic MPE who underwent high frequency ultrasound‐guided pericardiocentesis. After the procedure, all patients were followed for 90 days with the main purpose of detecting procedure success, procedure‐related complications, and recurrent PE.

**Results:**

Procedure success rate for pericardiocentesis was 100%. All patients were placed in the sitting position with their left hands extended above the heads. An apical puncture approach was performed in all cases (100%). The mean duration of catheter drainage was 8.1 ± 3.2 days. The mean initial amount of pericardial fluid drained was 956.3 ± 687.5 ml. Overall, six patients (11%) had recurrent PE; 3 (6%) had repeated percutaneous pericardiocentesis. There was no major complication and minor complications occurred in four patients (8%).

**Conclusion:**

This novel in‐plane high frequency US‐guided apical approach has several advantages for percutaneous pericardiocentesis of MPE: performed in the sitting position; a benefit for patients with orthopnea; a maximum inserted wide angle to prevent damage to the myocardium; local enlargement of the PE region; high procedure success rate of pericardiocentesis; and excellent clinical outcomes.

## 1 INTRODUCTION

Neoplastic involvement of the heart and pericardium has been reported in up to 21% of oncology patients.^1^ Malignant pericardial effusion (MPE), commonly found in cancer patients, can be associated with a poor prognosis.,2 Traditionally, percutaneous pericardiocentesis, which is less invasive than a surgical approach, is a valuable approach in the treatment of patients with moderate or large symptomatic MPE.^4^, ^5^, ^6^, ^7^ Described and developed by the Mayo Clinic,^7^ pericardiocentesis under echocardiographic guidance has been demonstrated to be well‐accepted, safe, and effective. However, during this echo‐assisted procedure, the operator memorizes the optimal needle trajectory and the needle is not under continuous visualization in real‐time as it enters the anterior aspect of pericardial space.^4^, ^8^, ^9^ Imprecise needle targeting can increase the probability of inadvertent injury to the heart, vascular structures and nearby organs.^4^, ^5^, ^6^, ^7^, ^8^ Therefore, difficulties associated with needle targeting during effusion drainage can contribute to the complication rates reported in the literature.^8^

Various nonconventional techniques of pericardiocentesis including in‐plane linear array ultrasound (US)‐guided pericardiocentesis for small children during the postoperative period,^9^high frequency US‐guided pericardiocentesis by the medial‐to‐lateral parasternal approach,^10^ and computed tomography (CT)‐guided pericardiocentesis (CTP) with poor echocardiographic acoustic windows and loculated pericardial effusions^11^ have been described in the literature. High frequency US (HF‐US) is largely used to guide percutaneous thoracic interventions.^12^ Due to its ability to directly visualize the superficial part of the pericardium, intercostal vessels and myocardium,^10^, ^14^ it is possible that HF‐US might serve as an alternative tool during PE drainage.^10^, ^11^, ^13^ So far, pericardiocentesis is performed in a supine or semi‐supine position.^8^, ^9^, ^10^, ^11^, ^12^ Nevertheless, patients with severe dyspnoea cannot lie flat and become orthopneic.^14^ A pericardiocentesis， performed in a supine or semi‐supine position, may not be appropriate for a patient with MPE in the acute symptomatic phase, due to orthopnea.

We hypothesized that an in‐plane HF‐US‐guided apical approach, performed in the sitting position, would provide additional benefits in terms of feasibility and safety for draining MPE. In this study, we have described our clinical experience using this novel approach. To our knowledge, this is the first study to report, in detail, a novel apical approach for draining MPE in the sitting position.

## 2 MATERIALS AND METHODS

### 2.1 Patients

All patients who were treated for MPE by percutaneous pericardiocentesis with an in‐plane apical approach performed in the sitting position from November 2015 to June 2019 were retrospectively enrolled. MPE was defined as the presence of atypical or overtly malignant cells in pericardial effusion or the drainage of exudate effusion in patients with malignancy, in the absence of other causes, such as tuberculosis or post‐operative effusion. Patients were included if they had moderate or large symptomatic MPE, and underwent primary percutaneous pericardiocentesis. All patients had symptomatic MPE confirmed on echocardiography. MPE was classified based on its size as mild (<10 mm), moderate (10–20 mm), or large (>20 mm).^15^ We did not have on‐site cardiac surgery in our center. All procedures were performed by one experienced radiologist who had operated for more than 8 years in pericardiocentesis.

Exclusion criteria included prothrombin time of more than 22 s and platelet count less than 50 cells × 10^9^/L. Anticoagulated patients were also excluded. This retrospective observational study was approved by the Harbin Medical University Cancer Hospital Ethics Committee and written informed consent was obtained from all patients enrolled in this study.

### 2.2 Procedural preparations

**FIGURE 1:**
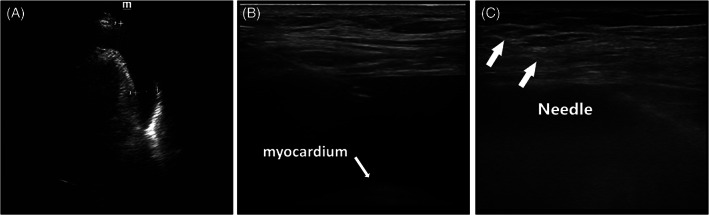
Representation of large symptomatic MPE in a 72‐year‐old man with a history of lung cancer. (A) Conventional echocardiogram performed before the procedure showed a large pericardial effusion. The maximal anterior depth was 29 mm. (B) The apical view was obtained with the high frequency probe and the depth adjustment was 4 cm. The needle is continuously visualized by a high frequency ultrasound while entering the pericardial space. (C) After the needle penetrated through the pericardial space, a j‐tipped guidewire was gently advanced through the needle

- Prior to the procedure, MPE was initially evaluated with a standard phased array probe for the size and needle insertion site of the PE (Figure 1(A)) as part of our standard practice. A Mindray DC‐8 ultrasound machine (Mindray, ShenZhen, China) with a cardiac phased array probe (1.0–5.0 MHz) and a high frequency probe (5.0–14 MHz) were used.
- After baseline cardiac US examination, the apical view was obtained with the high frequency probe (5.0–14 MHz) and the apical cardiac notch was visualized and the distance from the skin to the pericardium and effusion diameter were measured. An effusion of more than 1 cm in the apical window was considered suitable for the in‐plane pericardiocentesis technique.

### 2.3 Pericardiocentesis technique

**FIGURE 2:**
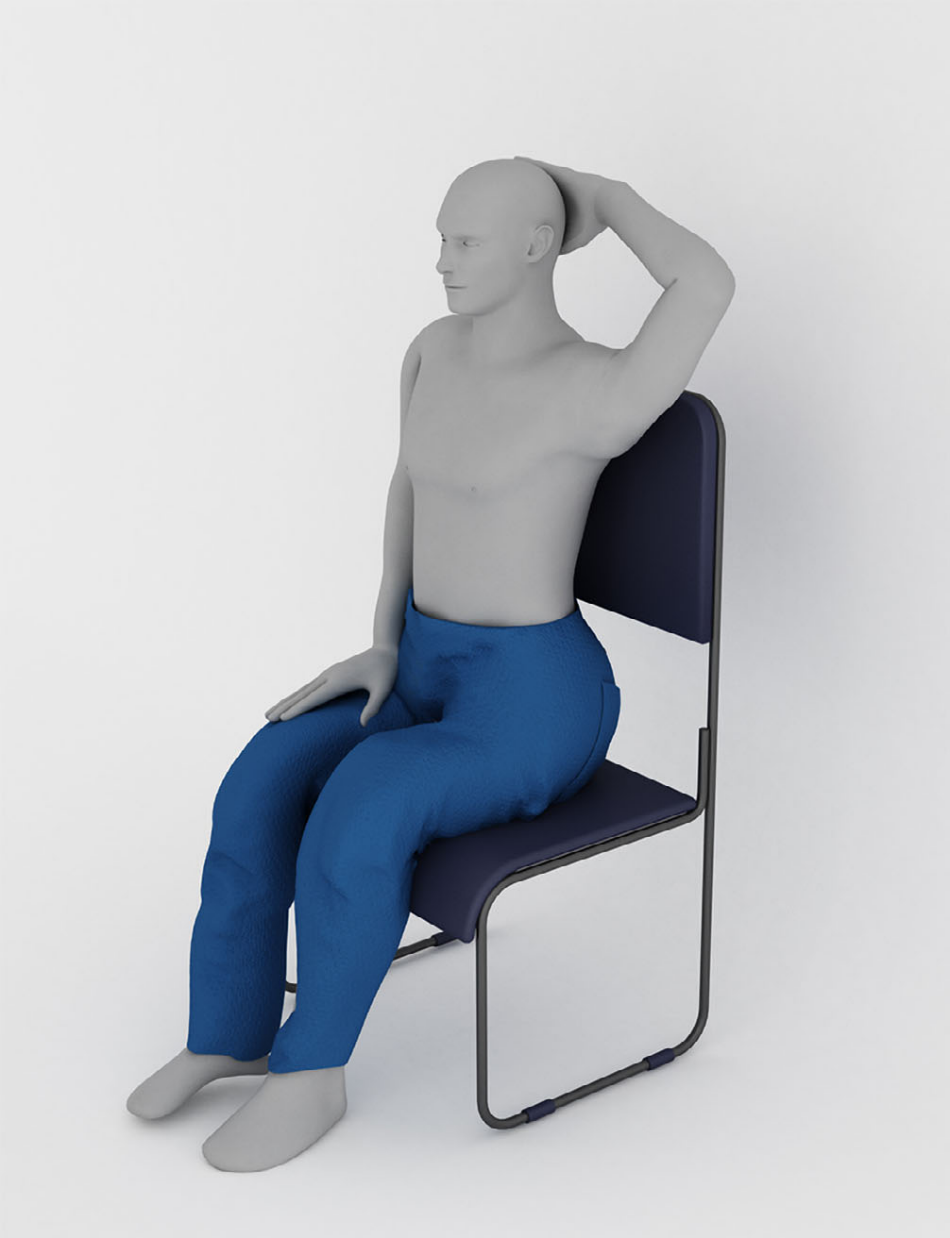
Schematic of the patient in the sitting position during pericardiocentesis. (1) The patient was allowed to sit on chair with a backrest. (2) The patient's stability was ensured (e.g., with aid from an assistant or nurse). (3) The patient's left arm was extended above the heads to stretch the intercostal space

**FIGURE 3:**
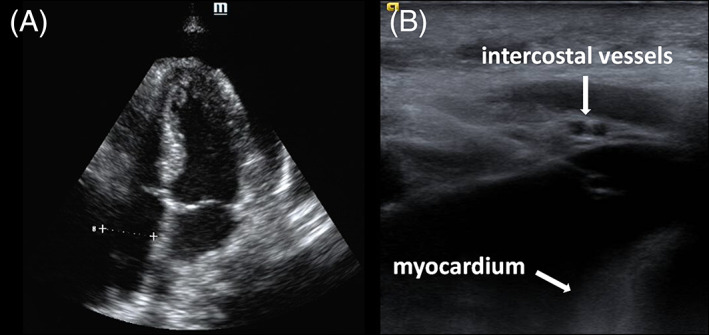
Moderate symptomatic MPE in a 46‐year‐old woman with a history of breast cancer. (A) Conventional echocardiogram performed before the procedure showed a moderate pericardial effusion. The maximal anterior depth was 19 mm. (B) The needle is continuously visualized by a high frequency ultrasound while entering the pericardial space. A safe approach to needle advancement under high frequency ultrasound guidance while continuous visualization of the needle insertion, superficial part of the pericardium, intercostal vessels and myocardium

**FIGURE 4:**
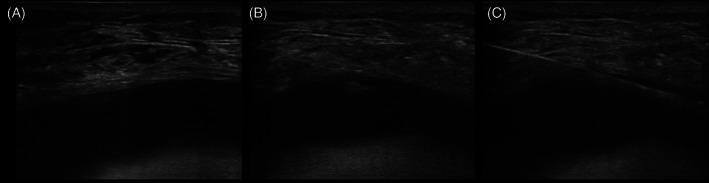
Presentation of the maximum inserted wide angle approach. (A) illustrates a moderate PE, the maximal anterior depth was 14 mm. (B) To prevent damage to the myocardium, the maximum inserted wide angle between the probe and the needle was chosen. The maximum wide angle allowed a better visualization of the needle in the short space between the probe and the pericardium sac. (C) The needle and the guidewire into the pericardial cavity was approximately parallel to the myocardium

- We allowed the patient to assume the fully upright sitting position, and placed the patient's left arm extended above the heads to stretch the intercostal spaces (Figure 2). If the patient had a problem with his left arm and could not extend above his head, the patient would be excluded. An US machine was positioned to the right of the patient, and the operator on the left, allowing a direct view of the US screen after optimal US setting adjustment. Vital signs and pulse oximetry were monitored during the procedure.
- Depth of the rectangle on the US screen and focus position, using the high frequency probe, was adjusted so that only the PE and the left ventricle were visible.
- The apical US examination, using the high frequency probe to identify the internal intercostal vessels(Figure 3), ribs, lung, pericardial effusion, and myocardium (left ventricle), was performed and the optimal puncture path for drainage was chosen. The optimal puncture path was defined as the pathway which had the minimum distance between the needle insertion site and the pericardial sac and avoided essential structures.
- All procedures were performed under sterile conditions. The needle insertion site was then anesthetized with 2% lidocaine from the skin down to the cardiac pericardium along the planned puncture path (Video S1). The high frequency probe was covered with an aseptic sheath.
- The operator sat facing the patient and held the probe in the left hand while smoothly advancing the needle (18‐gauge, 7 cm length, SCW MEDICATH Ltd., ShenZhen, China) with the right hand, toward the fluid collection. The needle connected to a syringe was advanced 'in plane' with the probe and clearly identified in its puncture path as it approached the pericardial sac (Figure 1(B)).
- To prevent damage to the myocardium, under US guidance the needle was inserted with a maximum wide angle (45^。^‐ 90^。^) between the probe and the needle. The maximum inserted wide angle allowed a better visualization of the needle in the short space between the probe and the pericardium sac. Meanwhile, the needle into the pericardial cavity was approximately parallel to the myocardium (Figure 4).
- After the needle penetrated through the pericardial space, the operator then stopped the insertion of the needle and checked for aspirated fluid with the connected syringe.
- A j‐tipped guidewire (0.035‐inch, 45 cm length, SCW MEDICATH Ltd.) was gently advanced through the needle while observing the needle tip on the ultrasonic screen, and the needle was then removed (Figure 1(C)). The location of the guidewire in the pericardial space was identified by observing it moving freely under ultrasound.
- A dilator was then inserted along the guidewire. Finally, a central venous catheter (7F, 20 cm length, SCW MEDICATH Ltd.) with double lumen catheters was inserted into the pericardial space over the guidewire after the dilator was removed.
- The pericardial fluid was aspirated with a syringe, and aspiration was repeated every 6 h. Routine pericardial catheter care instructions include meticulously cleaning the capped end of the pericardial catheter, injecting 5 ml of sterile 0.9% saline to flush the catheter of debris, aspiration of the entire volume of pericardial fluid, and injection of 3 ml sterile 0.9% saline to flush and lock the catheter.^4^

Drained fluid (80 ml) was drawn for cytology analyses. To assess catheter position and periprocedural complication (e.g., pneumothorax, hemorrhage), a post‐procedure chest X‐rays was regularly performed. The drainage catheter remained in place until the daily drained fluid reached <25 ml, and follow‐up echocardiography was performed to ensure no significant residual PE was present at that time.

### 2.4 Definitions and statistical analysis

After the procedure, all patients were followed for 90 days with the main purpose of detecting procedure success, procedure‐related complications, and recurrent PE. Recurrent PE was defined as reaccumulation of fluid within 90 days, documented by echocardiography. Any management of such recurrence was collected. Procedure success was defined as successful initial percutaneous access into the pericardial space with pericardial fluid via the catheter and subsequent symptomatic relief. Complications were reviewed and categorized into major and minor complications in accordance with the guidelines published by the Society of Interventional Radiology(SIR).^16^

All data calculations were performed with the JMP‐8 software (SAS Institute, Cary, USA). Continuous variables were reported as the mean ± SD. Categorical variables and complications were reported as counts and percentages and compared by the Fisher's exact test. A two‐tailed p value <.05 was considered statistically significant.

## 3 RESULTS

### 3.1 Patient characteristics

From November 2015 to June 2019, we selected 53 consecutive patients with moderate or large symptomatic MPE who underwent continuous HF‐US‐guided pericardiocentesis. The patients' clinical characteristics are summarized in Table 1. Of all patients with symptomatic MPE, 27 (51%) had moderate PE (<2 cm), and 26 patients (49%) had large PE. In most cases (43, 81%), hemodynamic instability (i.e., cardiac tamponade) formed the main indication for the pericardiocentesis.

**TABLE 1:** The patients' clinical characteristics (*N* = 53)

Parameter	Frequency
Age, years	54.3 ± 8.8
Sex	
Male	26(49)
Female	27(51)
BMI (kg/m^2^)	24.6 ± 3.7
Cancer type	
Lung	38(72)
Breast	10(19)
Mediastina	3(6)
Gastrointestinal	1(2)
Bladder	1(2)
Prior history of chest radiotherapy	29(55)
Prior history of chest surgery	14(26)
MPE size	
Moderate (10–20 mm)	27(51)
Large (>20 mm)	26(49)
Loculated pericardial effusion	0(0)
Hemodynamic instability	43(81)
Orthopnea	24(45)
Cardiac tamponade	9(17)

*Note*: Values are mean ± *SD* or *n* (%).

### 3.2 Procedural characteristics

Procedural success rate for pericardiocentesis was 100%. An apical puncture approach was performed in all cases (100%). The mean duration of catheter drainage was 8.1 ± 3.2 days. Most patients had extended catheter drainage for 3 to 8 days (32 patients, 60%); 21 (40%) required 9 to 14 days. The mean initial amount of pericardial fluid drained was 956.3 ± 687.5 ml (range: 95 to 2700 ml). The most common type of pericardial fluid aspirated was macroscopically hemorrhagic (42 patients [79% of all cases]), followed by serous (11 patients). Cytologic analysis of the pericardial fluid had a positive finding for malignant cells in 36 patients (68% of all cases). Although 17 patients (32%) were negative for malignant cells, exudate effusion was drained from the patients with malignancy and without other causes, such as tuberculosis or postoperative effusion. Therefore, these patients were regarded as having MPE. Certain variables such as blood pressure, heart rate before and after the procedure could not be retrieved from the records.

### 3.3 Clinical outcomes

At 90 days follow‐up, there were no patients lost. Extended catheter drainage was associated with recurrent PE rates of 13% and 10% when the indwelling catheter was left for 3 to 8 days or 9 to 14 days, respectively. Overall, six patients (11%) had recurrent PE; 3 (6%) had repeated percutaneous pericardiocentesis. The remaining three patients were followed up clinically without any further treatment of the recurrent PE. There was no major complication such as injury to an intercostal vessel, pneumothoraces and chamber lacerations observed in this study. Minor complications occurred in four patients (8%)(Table S1).

## 4 DISCUSSIONS

In an era of multimodality imaging in cardiology, there has been substantial improvement in the application of pericardiocentesis.^4^, ^5^, ^6^, ^7^ Electrocardiogram‐guided^7^ and CT‐guided^17^ techniques have been described. Much of this improvement has been mainly through echocardiography with a high degree of success and a low level of complications.^8^, ^19^, ^20^ Echocardiography, above all, is probably best when using a probe‐mounted needle,^19^ and is widely available and less time‐consuming than CT. However, it can be more complex in several types of patients, such as those recently submitted to cardiothoracic surgery, as well as those with poor echocardiographic acoustic windows.^12^, ^21^, ^22^ CT‐guided pericardiocentesis^12^, ^18^ is also a valuable and practical option with the advantage of detailed three‐dimensional imaging, which allows the physician to better evaluate needle direction and tip positioning. It should be acknowledged, however, that the main limitations of CT are probably its availability and prolonged procedural durations, reported to be a median time of 65 min in one series.^11^ Similarly, CTP may not be suitable for patients with orthopnea caused by pericardial effusion. Even if the patient can lie down, it is difficult for the patient to persist for such a long time due to the long time procedural durations of CTP.

In this study, we report our preliminary clinical experience on a novel in‐plane HF‐US‐guided apical approach for draining MPE, performed in the sitting position, which was easy to perform with the 100% success rate and no major complications. The most important characteristics of this apical approach are continuous visualization of the PE anterior aspect by HF‐US; a maximum inserted wide angle to prevent damage to the myocardium and local enlargement of the PE region. This study also showed that upright seated positioning was an acceptably safe approach for draining MPE.

Despite the well‐accepted application of echocardiographic‐ guided pericardiocentesis, major complication such as injury to an intercostal vessel, pneumothoraces, chamber lacerations and pleuropericardial shunts can still be potential complications associated with pericardiocentesis.^20^, ^23^, ^24^One of the most important reasons of major complication is imprecise needle targeting which can increase the probability of inadvertent injury to structure of the heart and nearby organs.^4^, ^5^, ^6^, ^7^, ^8^ Current conventional echocardiographic‐guided techniques often result in imprecise needle targeting.^8^, ^9^, ^20^ To overcome this problem, the HF‐US guidance technique was devised.^10^, ^11^ It is of note that high frequency linear array ultrasound is useful to evaluate the most superficial part of the soft tissues whereas lower frequency ultrasound are better at imaging deep tissues.^10^, ^11^, ^14^ The procedural success rate for pericardiocentesis is 100% in our study and is similar to other studies (91.7%–99%)^12^, ^14^, ^25^ possibly due to continuous visualization of the needle insertion, superficial part of the pericardium, intercostal vessels and myocardium, which are important to safely reach the pericardial space.

At the present time, the apical and subxiphoid approaches are the two most often used for pericardiocentesis.^8^, ^26^ The literature describes that the apical approach is the preferred location in 69%–79%^8^, ^20^of the cases, compared with the subxiphoid approach preferentially selected. The procedure success rate for pericardiocentesis in such cases was 97%–99%. In such serieses, all the patients were placed in the supine or semireclining position. Safe patient positioning involves balancing procedural comfort and optimal procedural setting against the risks related to the patient position.^26^ Ibrahim et al. reported successful echocardiography and fluoroscopy guided postoperative pericardiocentesis with the patient in slightly semi‐seated position.^27^ Mitsuda et al. reported successful US‐guided peripherally inserted central catheter (PICC) in a patient with congestive heart failure in the sitting position.^28^ This technique of inserting a drainage in a patient who has assumed the sitting position is simple and feasible. In our retrospective observational study, all patients were placed in the fully upright sitting position with their left hands extended above the heads. Twenty‐four patients who had orthopnea, required continuous oxygen, and had difficulty transferring to the surgical chair from wheelchair, and it might be preferable to perform pericardiocentesis with the patient in his or her own wheelchair. Due to this fully upright sitting position, a large amount of pericardial effusion accumulated to the inferoanterior part of the apex. An apical puncture approach was performed in all cases (100%) without difficulty. Such a position was proven acceptable and effective, with excellent complementary in the use of apical approach.

In a retrospective study of 110 patients with cardiac tamponade, Vayre et al.^24^ reported 11 right ventricular punctures (10%). The subxiphoid approach was performed in almost all cases (109 of 110 patients), however we think that the optimal site for needle insertion corresponds to the point where the fluid accumulation has the maximum thickness, and the pericardial space is closest to the thoracic wall and probe; in our experience, the maximum PE thickness can be reached by an apical approach more often than by a subxiphoid approach. As in the Mayo Clinic study,^7^ the preferred location was the chest wall in 79% of the cases, where the fluid accumulation is maximum can reduce the probability of chamber lacerations.

Danielle et al.^22^ reported a pericardiocentesis procedural success rate of 99% and 194 patients (91.5%) with large PE(>20 mm). In the present study, however, 51% of all apical approaches were performed in patients with moderate PE (27 of 53 patients). Our procedure success rate was 100% with no major complications related to myocardium injury. Moderate PE (10–20 mm) means a shallow space between the entry site and myocardium (Figure 3(A)). In such situations to prevent damage to the myocardium, the maximum inserted wide angle between the probe and the needle was chosen. The maximum wide angle allowed a better visualization of the needle in the short space between the probe and the pericardium sac (Figure 3(B)). Meanwhile, the needle into the pericardial cavity was approximately parallel to the myocardium (Figure 3(C)).

It must be highlighted that local enlargement of the PE region using HF‐US was another important characteristic of this technique. Through the use of HF‐US, we can clearly show the structures about 4 cm deep from the skin, which basically includes the chest wall, pericardial effusion, myocardial and other main structures, which are also the focus of pericardiocentesis. A moderate PE fluid accumulation this small does not give much space for procedural error during needle insertion. The present technique admits local enlargement of the PE region during the entire needle insertion, potentiating more precise entry into the small pericardial space. The real advantage of HF‐US guidance of pericardiocentesis is a significant reduction in the myocardium punctures/ lacerations. In a recent position statement of the European Society of Cardiology, the importance of having a low rate of cardiac perforations is also noted.^29^

Recurrent PE is considered a weakness of percutaneous pericardiocentesis. Extended catheter drainage has reduced this problem. In our study, six patients (11%) had recurrent PE, and three patients (6%) had a second percutaneous pericardiocentesis. This result was comparable to other similar studies.^20^, ^23^, ^26^

### 4.1 Study limitations

There are several limitations to this study. First, it is a single‐center study and the number of patients was relatively small. There may also be an element of selection bias. Second, the results of present study might not be generalizable to all physicians. Third, during the study period, patients with other approaches of pericardiocentesis were not included in our study; the comparison between our approach and common approach such as subxyhoid or transapical is therefore unknown. Further multicenter prospective randomized study comparing this technique with other techniques is warranted to confirm the initial promising results obtained in this single‐center study.

Fourth, this approach is not suitable for lateral or anteriorly and small pericardial effusion. Fifth, we did not track the number of cases that were performed with conventional pericardial puncture method during the study period.

## 5 CONCLUSIONS

Findings in present study suggest that this in‐plane HF‐US‐guided apical approach has several advantages for percutanefous pericardiocentesis of MPE: performed in the sitting position; a benefit for patients with orthopnea; a maximum inserted wide angle to prevent damage to the myocardium; local enlargement of the PE region; high procedure success rate of pericardiocentesis; and excellent clinical outcomes. This apical approach, performed in the sitting position, appears to be a novel, safe, effective and feasible technique in treating MPE.

## CONFLICT OF INTEREST

The authors declare that there is no conflict of interest.

## Supporting information

**Table S1** Complications of Percutaneous Pericardiocentesis (*N* = 53)

Video S1
