# Supplementary material for: High frequency ultrasound‐guided pericardiocentesis performed in the sitting position: A novel apical approach
Source: Clin Cardiol. 2021 Jun 8;44(8):1106–12. doi: 10.1002/clc.23657 (PMC8364732; doi:10.1002/clc.23657)
Supplement: Supplementary file 1 — Table S1 Complications of Percutaneous Pericardiocentesis (N = 53) [file CLC-44-1106-s001.docx]

Supplemental Table 1. Complications of Percutaneous Pericardiocentesis (N=53)

| **Parameter** | **Frequency** |
| --- | --- |
| Minor complications |  |
| Vasovagal response | 2(4) |
| Ventricular tachycardia | 1(2) |
| Pericardial catheter occlusion | 1(2) |
| Major complications | 0 |

Values are n (%).
